# Lymphoplasmacytic lymphoma and marginal zone lymphoma involving bone marrow: A diagnostic dilemma. Useful clinicopathological features to accurate the diagnosis

**DOI:** 10.1002/jha2.573

**Published:** 2022-10-05

**Authors:** Patricia García‐Abellás, Ana Ferrer Gómez, Diego Bueno Sacristán, Miguel Piris Villaespesa, María Talavera Yagüe, María Eugenia Reguero Callejas, Mónica García‐Cosío

**Affiliations:** ^1^ Department of Pathology Ramón y Cajal Universitary Hospital Madrid Spain; ^2^ Department of Hematology Ramón y Cajal Universitary Hospital Madrid Spain; ^3^ Department of Genetics Ramón y Cajal Universitary Hospital Madrid Spain; ^4^ Head of Hematopathology Department Ramón y Cajal Universitary Hospital; Alcalá University, Madrid, Spain; Instituto Ramon y Cajal de Investigacion Sanitaria, Madrid, Spain; CIBERONC Madrid Spain

**Keywords:** bone marrow, diagnosis, lymphoplasmacytic lymphoma, marginal zone lymphoma, Waldenström macroglobulinemia

## Abstract

Lymphoplasmacytic lymphoma (LPL) and marginal zone lymphoma (MZL) frequently infiltrate the bone marrow with similar histologic and immunohistochemical characteristics posing diagnostic problems. Bone marrow biopsy specimens from 25 LPL and 16 MZL have been studied, correlating with clinical, laboratory parameters and the MYD88_p.L265P mutation. Paratrabecular and interstitial infiltration pattern, serum IgM paraprotein levels, and MYD88_p.L265P mutation were significantly more frequent in LPL. Nodular or intrasinusoidal pattern with lymphocytosis and splenomegaly were associated with MZL diagnosis. Different clinical and histological parameters should be collected when LPL or MZL is suspected in bone marrow biopsy specimens.

## 1 INTRODUCTION

Lymphoplasmacytic lymphoma (LPL) is a B‐cell neoplasm composed of small lymphocytes, lymphoplasmacytoid, and plasma cells, usually involving the bone marrow. In a small subset of patients affects lymph nodes and spleen. Waldenström macroglobulinemia (WM) is defined as LPL with bone marrow involvement and IgM monoclonal gammopathy. Symptoms are usually related with bone marrow infiltration (anaemia, thrombocytopenia or leukopenia) and paraprotein deposition (cryoglobulinemia, neuropathy, diarrhoea, coagulopathy). Marginal zone lymphomas (MZL) are indolent B‐cell lymphomas characterised by the proliferation of B cells from the marginal zone of B‐cell follicles. This lymphoma includes three different entities, extranodal MZL of MALT type (EMZL), splenic lymphoma (SMZL), and nodal MZL (NMZL). Plasmacytic differentiation is frequent and differentiation from LPL may be challenging, especially in cases with paraproteinemia. Morphologic, immunophenotypic, and clinical features are necessary to make a certain diagnosis, since new pathogenic mechanisms and therapeutic strategies have been proposed [1, 2]. MYD88_p. L265P gene mutation, present in more than 90% of LPL, was described initially by Treon et al. as a useful tool to establish an accurate diagnosis [3]. Nevertheless, this mutation is not specific and has also been described in a small percentage of MZL[4, 5, 6].

As LPL and MZL can infiltrate the bone marrow with similar histologic and immunohistochemical characteristics, several studies defining the distinctive features of each neoplasia have been published with no matching results [7, 8, 9, 10].

The aim of the present article is to study the clinical and bone marrow histological features from series of LPL and MZL cases, highlighting the distinctive features that could help in the differential diagnosis.

## 2 MATERIAL AND METHODS

### 2.1 Patient selection

A retrospective review of bone marrow biopsy specimens with the diagnosis of LPL and MZL from 2017 to 2021 from the Pathology Department files of Ramón y Cajal Universitary Hospital was performed. In total, 25 patients with LPL and 16 patients with MZL were included (eight SMZL and eight NMZL).

### 2.2 Histological and immunohistochemical study

Bone marrow trephine biopsies were reviewed by two pathologists (M.G.C and P.G.A). Histopathological infiltration patterns in the bone marrow were categorised as intrasinusoidal, diffuse, paratrabecular, nodular, and interstitial. A neoplastic infiltrate was considered paratrabecular when the contact surface of the infiltrate with the trabecular bone was larger than the maximum diameter perpendicular to the bone; interstitial if solitary or groups of the neoplastic cells were intermingled with normal bone marrow cellularity; nodular when clearly defined focal infiltrates were found in an intertrabecular location and diffuse when confluent areas of infiltration with a loss of hematopoietic elements and fat spaces are observed. Percentage of each pattern, immunophenotype profile, quantification of mast cells, plasma cells, or the presence of immunoglobulin light chain restriction was also recorded. Immunohistochemical analysis was performed with the following monoclonal antibodies: CD20 (clone L26, Agilent), CD3 (polyclonal, Agilent) CD79 (clone JBC117, Agilent), CD138 (clone MI15, Agilent), tryptase (clone AA1, Agilent), ƙ light chains (polyclonal, Agilent), ʎ light chains (polyclonal, Agilent), CD25 (clone 4C9, Leica biosystems), and CD5 (clone 4C7, Agilent). All of them were done on an OMNIS (Agilent) automated stainer, except for CD25, which was performed on the BOND III systems (Leica Biosystem), according to the manufacturer's instructions. Appropriate external positive controls were used.

The cut‐off value for defining lymphoma with plasmacytic differentiation was CD138 expression in the neoplastic cells higher than 10% [7]. We considered an arbitrary cut‐off value for defining overexpression of mast cells as 10% or higher of the global bone marrow cellularity.

### 2.3 Clinical data

Clinical and laboratory parameters such as age, gender, the presence of lymphocytosis (≥5 × 10 [9]/L), lymphadenopathy, splenomegaly, and elevated serum IgM paraprotein were collected from electronic clinical records.

### 2.4 Statistical analysis

Continuous variables were expressed as mean ± standard deviation and qualitative variables were expressed as number and percentage. Continuous variables were compared using Student's *t* test or the Mann–Whitney *U* test according to their distribution, and categorical variables were compared using the chi‐squared test or the Fisher's exact test as appropriate.

### 2.5 Molecular analysis

MYD88_p. L265P mutation status was determined using real‐time allele‐specific polymerase chain reaction and Sanger DNA sequencing from diagnostic bone marrow aspirates and/or peripheral blood.

## 3 RESULTS

Clinical, histological and laboratory parameters are described in Table 1. A summary of the clinical and pathological features of lymphoplasmacytic lymphoma and marginal zone lymphoma with statistical significance is defined in Table 2.

**TABLE 1:** Clinical, histological, and laboratory parameters in lymphoplasmacytic lymphoma and marginal zone lymphoma cases

Case	Gender	Age	Diagnosis	Lymphadenopaties	Splenomegaly	Lymphocytosis	IgM (mg/dl)	MYD88	% B.M	% PT	% D	% N	% IS	% I	% CD138	LCR	% tryp	CD25	CD5
1	F	74	LPL	Yes	No	No	1350	pos	60	50	30	20	0	0	12	nr	12	No	No
2	M	50	LPL	No	Yes	No	2450	pos	60	5	0	10	5	80	20	K	7	No	No
3	M	78	LPL	No	Yes	No	3230	pos	30	30	0	10	0	60	12	K	5	No	No
4	F	57	LPL	Yes	No	No	1040	pos	20	40	0	20	0	40	6	nr	2	No	No
5	M	64	LPL	No	No	No	1680	pos	30	0	0	70	0	30	5	K	2	Yes	No
6	M	74	LPL	Yes	Yes	No	1590	pos	90	0	80	0	0	20	20	K	2	No	No
7	M	38	MZL	No	Yes	Yes	nd	nd	15	0	0	90	10	0	5	nr	1	No	No
8	M	76	MZL	No	Yes	Yes	nd	nd	50	0	10	10	0	80	4	nr	2	No	No
9	F	81	MZL	Yes	Yes	Yes	340	nd	70	0	100	0	0	0	5	nr	2	No	No
10	M	57	LPL	Yes	Yes	No	96	pos	30	10	80	0	0	10	18	K	10	Yes	No
11	M	71	LPL	Yes	No	No	958	pos	30	20	0	0	0	80	10	K	15	No	No
12	F	78	LPL	Yes	No	No	1600	pos	10	0	0	0	0	100	3	nr	1	No	No
13	M	76	MZL	Yes	No	No	177	neg	25	10	0	10	10	70	5	K	5	No	No
14	M	73	MZL	No	Yes	Yes	nd*IgG 2600	neg	80	0	10	80	0	10	7	K	5	No	No
15	F	69	MZL	No	Yes	Yes	59	neg	80	0	0	90	5	5	10	L	5	No	No
16	M	52	MZL	Yes	Yes	Yes	7	neg	90	0	0	0	0	100	3	nr	1	No	No
17	F	83	MZL	Yes	Yes	Yes	22	nd	7	0	0	90	5	5	5	K	3	No	No
18	M	70	MZL	No	Yes	Yes	nd	nd	15	0	0	0	100	0	1	nr	1	No	No
19	F	87	MZL	No	Yes	Yes	461*IgA 868	neg	5	20	0	0	30	50	15	L	3	No	No
20	F	82	MZL	No	Yes	No	29	nd	15	0	10	60	15	15	10	nr	5	No	No
21	M	72	LPL	No	No	No	274	nd	70	20	0	0	20	60	70	L	5	No	No
22	F	81	LPL	No	No	No	300	nd	10	0	0	10	0	90	10	K	1	No	No
23	F	75	LPL	No	Yes	Yes	244	pos	30	0	0	0	0	100	5	K	3	Yes	No
24	M	68	LPL	Yes	No	No	3600	pos	7	0	0	10	0	90	12	K	5	No	No
25	M	83	LPL	No	No	Yes	346	pos	10	0	0	0	0	100	5	K	1	No	No
26	F	86	LPL	No	No	No	282*IgG 1730	neg	20	30	0	30	10	30	10	K	6	Yes	No
27	M	62	LPL	No	Yes	No	4500	pos	60	10	0	0	0	90	5	K	1	No	No
28	M	76	LPL	Yes	No	No	3760	pos	70	40	40	20	0	0	20	K	7	No	No
29	M	51	LPL	No	Yes	No	2450	pos	10	10	0	80	0	10	2	K	1	Yes	No
30	F	67	MZL	No	No	No	91	neg	5	20	0	0	40	40	2	K	1	Yes	No
31	F	70	LPL	No	No	Yes	1660	pos	20	40	0	40	0	20	8	K	5	No	Yes
32	M	81	LPL	No	No	No	672	nd	70	0	0	0	0	100	20	L	10	Yes	Yes
33	M	69	LPL	Yes	No	No	336	pos	70	0	20	0	0	80	5	K	7	Yes	Yes
34	F	77	LPL	Yes	Yes	Yes	49	neg	60	0	0	0	0	100	2	nr	2	No	No
35	F	56	MZL	No	Yes	No	343	neg	15	0	0	20	60	20	3	nr	2	No	No
36	F	69	MZL	Yes	No	No	nd	nd	7	0	0	90	10	0	5	nr	5	Yes	Yes
37	F	82	MZL	No	Yes	No	17	nd	20	10	0	60	0	30	7	nr	3	Yes	No
38	F	58	MZL	Yes	No	Yes	nd	nd	30	0	0	95	0	5	2	nr	1	No	No
39	M	78	LPL	Yes	No	No	*IgG 3400	neg	5	100	0	0	0	0	7	K	2	Yes	No
40	F	77	LPL	No	No	No	2160	pos	10	10	0	0	0	90	10	K	1	No	No
41	F	71	LPL	No	No	Yes	1260	pos	20	10	0	0	0	90	7	K	5	No	No

Abbreviations: B.M., bone marrow; D, diffuse; LPL, lymphoplasmacytic lymphoma; MZL, marginal zone lymphoma; N, nodular; PT, paratrabecular; I, interstitial; IS, intrasinusoidal; LCR, light chain restriction; nd, not determined; nr, no light chain restriction.

*Monoclonal component not IgM. Normal levels (mg/dl): IgM 40–230; IgG 700–1600; IgA 70–400.

**TABLE 2:** Summary of the clinical and pathological features of lymphoplasmacytic lymphoma and marginal zone lymphoma cases with statistical significance

Variable	MZL *n* = 16	LPL *n* = 25	*p*‐Value
Gender	10 (63%)	10 (40%)	0.226
Age, mean (SD)	69.9 (13.3)	71.2 (9.6)	0.363
Lymphocytosis	10 (63%)	5 (20%)	0.009
Splenomegaly	12 (75%)	8 (32%)	0.011
Lymphadenopaty	6 (38%)	11 (44%)	0.549
IgM paraprotein	3 (30%)	22 (88%)	0.001
MYD88p.L265P	0 (0%)	19 (86.36%)	0.000
Infiltration, mean (SD)	33.1 (30.3)	36.1 (26.0)	0.367
Paratrabecular, mean (SD)	3.8 (7.2)	17.0 (23.5)	0.035
Diffuse, mean (SD)	8.1 (24.8)	10.0 (23.5)	0.404
Nodular, mean (SD)	43.4 (41.2)	12.8 (21.7)	0.003
Intrasinusoidal, mean (SD)	17.8 (27.8)	1.4 (4.5)	0.006
Interstitial, mean (SD)	26.9 (32.2)	58.8 (38.1)	0.008
CD138+, mean (SD)	5.6 (3.6)	12.2 (13.4)	0.062
Light chain			0.094
*K*	4 (67%)	19 (90%)	
*L*	2 (33%)	2 (10%)	
Light chain restriction	6 (38%)	21 (84%)	0.006
Mast cells triptase, mean (SD)	2.8 (1.7)	4.7 (3.8)	0.070
CD25	3 (19%)	8 (32%)	0.551
CD5	1 (6%)	3 (12%)	0.584

Abbreviations: LPL, lymphoplasmacytic lymphoma; MZL, marginal zone lymphoma.

**FIGURE 1:**
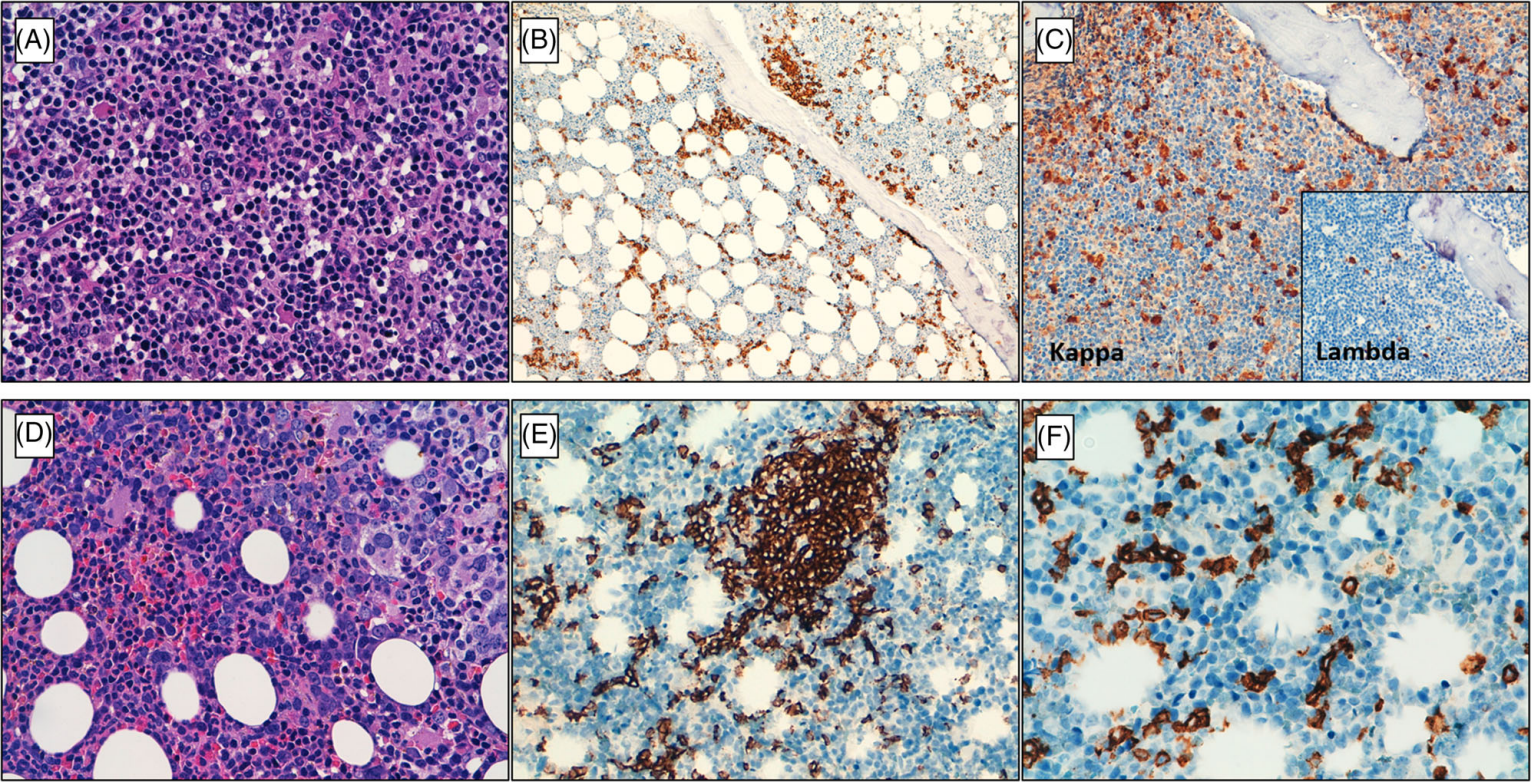
Bone marrow features of a representative  Lymphoplasmacytic lymphoma and marginal zone lymphoma.  (A; H&Ex40) Representative lymphoplasmacytic lymphoma. Small monomorphic lymphocytes admixed with plasmacytoid and plasmacytic cells. Staining for CD20 shows the paratrabecular and interstitial pattern of infiltration (B). Immunohistochemistry evidenced kappa immunoglobulin light chain restriction (C). Case 3. (D; H&E x40). Bone marrow features of a representative marginal zone lymphoma. Small and medium relatively monomorphic lymphocytes are present. Immunoperoxidase staining for CD20 highlights combination of nodular (E) and intrasinusoidal pattern of infiltration (F). Case 35.

Median percentage of bone marrow infiltration was quite similar in both entities: 36% in LPL and 33.1% in MZL. Paratrabecular and interstitial patterns of infiltration of the bone marrow were significantly more frequent in LPL (*p* = 0.035 and *p* = 0.008, respectively) whereas MZL showed more frequent intrasinusoidal and nodular involvement (*p* = 0.006 and *p* = 0.003, respectively). Plasmacytic differentiation was observed in 12.2% of LPL and in 5.6% of MZL cases. The percentage of cells with light chain restriction was higher in LPL compared to MZL cases (84% vs. 38%, respectively) (*p* = 0.006) (Figure 1). CD5 was expressed on B cells in three of the 25 (12%) LPL cases and in one of the 16 (6%) MZL cases. No significant differences were found. In four samples of LPL (16%), mast cells comprised ≥10% of the bone marrow cellularity. None of the MZL cases presented increasing number of mast cells.

Elevated serum monoclonal IgM component was detected in 88% LPL and in 30% MZL cases (*p* = 0.001). Interestingly, none of the MZL cases had an IgM higher than 1000 mg/dl. Lymphocytosis was presented in 63% of MZL and 20% of LPL cases (*p* = 0.009) and splenomegaly in 32% of LPL and 75% of MZL cases (*p* = 0.011). Otherwise, lymphadenopathy was present in 44% of LPL cases and in 38% of MZL cases, showing no statistically significant difference.

MYD88_p.L265P mutation was detected in 19 of 22 (86.36%) LPL studied cases and none of the 7 MZL studied cases (*p* = 0.000). In 18 of the 19 LPL cases in which MYD88_p.L265P mutation resulted positive, there was a serum IgM monoclonal component (94.73%). There was only one case nonsecretory. By contrast, in the MYD88_p.L265P mutation negative LPL cases, there was one case nonsecretory, one case non‐IgM (IgG), and in one case, the serum monoclonal component was both, IgM and IgG type.

## 4 DISCUSSION

B‐cell lymphoproliferative disorders with plasmacytic differentiation involving bone marrow represent a diagnostic challenge. Traditionally, it has been proposed that the presence of serum IgM paraprotein is useful for LPL diagnosis and could help in the differential diagnosis of MZL [11, 12]. However, IgM paraprotein can be seen in MZL in other B cell neoplasms.

In our study, the presence of elevated serum IgM monoclonal component was significantly more frequent in LPL than MZL. On the other hand, MZL presented significantly more blood lymphocytosis and splenomegaly. Lymphadenopathy was seen in both cases.

The histological patterns of bone marrow infiltration in each entity have been described by some authors as a very useful feature, with no concordant results. Bassarova et el. [8] have proposed the paratrabecular infiltration as the most characteristic architectural feature of LPL, although other authors have described intrasinusoidal and paratrabecular infiltration as characteristic of MZL[9, 10]. In accordance with other publications [13, 14], in our study, paratrabecular and interstitial patterns of infiltration were significantly higher in LPL, whereas intrasinusoidal or nodular infiltration was distinctive features of MZL.

It has been suggested that LPL and MZL also differ in the percentage of plasmacytic cells in the neoplastic infiltrate suggesting that a percentage of plasma cells higher than 10% could support LPL diagnosis [7]. In our cases, plasma cells were more abundant in LPL, without statistically significant difference. Moreover, according to Morice et al. [15], the percentage of cells with light chain restriction was significantly higher in LPL; hence we propose light chain restriction as a feature of LPL in bone marrow biopsies. Differentiating lymphomas with marked plasmacytic differentiation from plasma cell neoplasms can sometimes be a diagnostic challenge. Detection of CD19 and CD45 expression in neoplastic plasma cells of LPL and MZL can be useful [16].

Different publications remark that the immunophenotype of LPL and MZL is not very distinctive. Classically, LPL cells are negative for CD5 with frequent CD25 expression. However, some authors have described in some series the expression of CD5 in 43% of LPL cases [15]. In our series, no significant differences were found in the expression of CD5 or CD25.

Regarding the higher percentage of mast cells that have been described in LPL [17], our results conclude that this feature is relatively nonspecific and does not help in the differential diagnosis.

MYD88_p.L265P gene mutation was described in 2012 by Treon et al. as a useful tool to accurate the diagnosis of LPL [3]. However, the mutation that has been described in more than 90% of LPL cases has also been demonstrated less frequently in chronic lymphocytic leukemia/small lymphocytic lymphoma, follicular lymphoma, mantle cell lymphoma, and particularly in MZL [18]. Our study, in accordance with others, suggests that the presence of MYD88_p.L265P gene mutation could be an additional tool to provide a certain diagnosis. Interestingly, we found that only in the LPL cases in which MYD88_p.L265P mutation resulted positive, there was a serum IgM monoclonal component higher than 1000 mg/dl, suggesting a lower rate of the mutation in IgM cases with low monoclonal component and non‐IgM cases [19, 20].

The World Health Organization Classification of Haematolymphoid Tumours, in previous editions, suggested that “A small B‐cell lymphoid neoplasm with plasmacytic differentiation should be diagnosed when the distinction between LPL and MZL, is not always clear‐cut.” The results from the present study confirm that different clinical and histological parameters should be collected when LPL or MZL is suspected in bone marrow biopsy specimens, as it is suggested in the upcoming 5th edition of World Health Organization Classification of Haematolymphoid Tumours [21]. The recognition of paratrabecular and interstitial patterns of infiltration, the presence of plasmacytoid and lymphoplasmacytoid cells with light chain restriction, the presence of serum IgM monoclonal component higher than 1000 mg/dl, and the presence of MYD88_p.L265P mutation are findings that support LPL diagnosis. By contrast, blood lymphocytosis, splenomegaly, intrasinusoidal, and nodular pattern of bone marrow infiltration favor MZL diagnosis.

## AUTHOR CONTRIBUTIONS

Patricia García Abellás and Ana Ferrer Gómez performed the research. Mónica García‐Cosio designed the research study. Miguel Piris Villaespesa and María Talavera Yagüe contributed with the clinical information and the molecular analysis. Patricia García Abellás and Diego Bueno Sacristán analyzed the data. Patricia García Abellás and Mónica García‐Cosío Pique wrote the paper.

## CONFLICT OF INTEREST

There is no conflict of interest of any of the authors with the results of this study.

## FUNDING INFORMATION

This research received no specific grant from any funding agency in the public, commercial, or not‐for‐profit sectors.

## ETHICS STATEMENT

This study was approved by the Ramón y Cajal Universitary Hospital ethic committee and was conducted in accordance with the Declaration of Helsinki. Informed consent was obtained from all included patients.

## Data Availability

The data that supports the findings of this study are available in the references of this article.
